# Integrating morphological and deep learning approaches for the identification of economically important nematode genera in vineyards: *Mesocriconema* and *Xiphinema*

**DOI:** 10.2478/helm-2026-0002

**Published:** 2026-04-27

**Authors:** L. ÖZTÜRK, B. ŞİN

**Affiliations:** Independent researcher, 59200, Süleymanpaşa, Tekirdağ, Türkiye; Plant Protection Department, Faculty of Agriculture, Sakarya University of Applied Science, 54050, Sakarya, Türkiye

**Keywords:** plant parasitic nematodes, artificial intelligence, detection, image recognition

## Abstract

Accurate identification of plant-parasitic nematodes is fundamental to effective crop protection and the maintenance of soil ecosystem integrity. This study integrates morphological characterization with deep learning-based object detection to enhance diagnostic accuracy for economically important nematode genera, *Xiphinema* and *Mesocriconema*, associated with vineyards. Three advanced YOLO architectures [YOLO-NAS, YOLOv11, and Roboflow 3.0 (YOLOv8 architecture)] were trained and evaluated on a high-resolution annotated microscopic image dataset consisting of 961 images and 1.034 bounding-box annotations. Although the target nematode genera display considerable morphological variability and genetic divergence among populations, the present investigation focused on genus-level detection of *M. xenoplax* and *X. pachtaicum*. These two major ectoparasitic nematodes cause significant damage to grapevine root systems. Among the models tested, YOLOv11 achieved the highest detection accuracy, with a precision of 95.7 % and an mAP@50 of 93.2 %. YOLO-NAS exhibited comparable performance (mAP@50 = 92.7 %, precision = 93.1 %, recall = 84.9 %), while Roboflow 3.0 (YOLOv8 architecture) yielded satisfactory results (mAP@50 = 89.4 %), indicating its applicability for real-time diagnostic workflows. This integration of taxonomic expertise with deep learning represents a new methodological framework for nematode identification. All models exhibited rapid convergence and stable learning dynamics during training. The findings underscore the potential of YOLO-based frameworks as efficient, scalable, and reproducible tools that complement classical morphological and molecular identification, contributing to precision agriculture and sustainable nematode management strategies.

## Introduction

Nematodes are microscopic, soil organisms that cause significant yield losses in many agricultural crops worldwide. Among nematodes, the genera *Xiphinema* and *Mesocriconema* are of particular economic importance because they parasitize the root systems of grapevine, peach, walnut, and other perennial crops, leading to stunted growth and reduced yield (Ferris *et al*,. 2004; Deimi & Mitkowski, 2010). In vineyards, infestations by these ectoparasitic nematodes may reduce yield by more than 30 %, even at relatively low population densities (Pinkerton *et al*., 2004). Due to rapid population growth hidden in soil, early detection of these parasites is crucial for effective management.

Morphological and morphometric analyses have traditionally been the primary methods for identifying nematode taxa (Kumari & Lišková, 2009). These approaches, which rely largely on the examination of adult female specimens, are time-consuming and require advanced taxonomic expertise (Kumari *et al*., 2010). Misidentification at the genus level can lead to the use of an incorrect identification key; as a result, all species-level determinations and management decisions may become unreliable. Therefore, accurate genus-level identification represents the critical basis for all reliable identification and management procedures.

Although morphological examination remains the key component of nematode taxonomy, the growing demand for rapid, reproducible, and scalable diagnostic tools has accelerated the integration of computer vision and artificial intelligence (AI) into nematode identification workflows. Advances in these technologies have enabled the rapid detection of agricultural pests and diseases from microscopic imagery. Developed initially for real-time object detection, YOLO (You Only Look Once) models have been successfully adapted for detecting plant diseases, insect pests, and weed species with high accuracy and speed (Stark *et al*., 2022; Guo *et al*., 2023; Soeb *et al*., 2023; Aldakheel *et al*., 2024; Abid *et al*., 2024; Rodríguez Lira *et al*., 2024; Uygun *et al*., 2024; Wang *et al*., 2025). While previous studies mainly focused on the identification of species such as *Meloidogyne* by using YOLO models, this research uniquely integrates morphological identification with genus-level deep learning detection of *Xiphinema* and *Mesocriconema*, which are rarely addressed in vineyard nematology (Pun *et al*., 2022; Wang *et al*,. 2022). In particular, YOLO-based deep learning frameworks can classify nematodes at the genus level based on key morphological features such as body shape, tail structure, and stylet length, allowing for rapid and highly accurate recognition of nematode genera.

Compared to traditional microscopic examination, image-based approaches significantly reduce diagnostic time, minimize operator dependency, and improve reproducibility. Specimens identified at the genus level can then be distinguished at the species level using appropriate classical identification keys and morphometric criteria such as stylet length, body ratios, and vulva position. Thus, integrating image-processing–based pre-identification with classical taxonomic approaches provides a faster, more reliable, and more comprehensive diagnostic framework.

Recent progress in Al-driven image analysis has created new opportunities for nematology. The YOLO models offer distinct advantages for microscopic image analysis due to their lightweight structure, single-stage detection mechanism, and high inference speed (Redmon *et al*., 2016; Bjerge *et al*,. 2023).

The present study integrates morphological characterization with deep learning–based object detection to identify economically important nematodes at the genus level associated with vineyards. While the model targets genus-level discrimination rather than species-level resolution, it provides a practical diagnostic framework that complements, rather than replaces, classical morphological and molecular approaches. Three YOLO-based models (YO-LO-NAS, YOLOv11, and Roboflow 3.0 [YOLOv8 compatible]) were trained and evaluated on an annotated microscopic image dataset containing 961 images and 1.034 bounding box annotations. Specifically, we (i) assessed each model’s accuracy, precision, and recall, and (ii) demonstrated the potential of these models for automated nematode diagnostics. This framework highlights how artificial intelligence can complement morphological methods, providing a rapid, scalable, and reproducible approach to nematode identification and sustainable pest management. As far as is known, this is among the first studies to implement and compare YOLO-NAS, YOLOv11, and Roboflow 3.0 (YOLOv8 compatible) models for genus-level nematode detection from microscopic imagery.

## Material and Method

### Soil Sampling, Nematode Recovery Identification

To evaluate the genus-level detection performance of YOLO models, two of the most common nematode species found in Turkish vineyard soils were selected as representative taxa: *Mesocricone-ma xenoplax* (genus *Mesocriconema*) and *Xiphinema pachtaicum* (genus *Xiphinema*). A total of 961 high-resolution microscopic images were compiled to develop a dataset for the detection of *Mesocriconema* and *Xiphinema* using deep learning–based object detection models. The images were obtained from permanent nematode slides prepared under laboratory conditions.

Permanent slides were prepared from nematodes extracted from vineyard soil samples using the modified Cobb’s sieving and centrifugal flotation techniques (Jenkins, 1964; Brown & Boag, 1988). Individual nematodes were heat-killed and fixed in a double-strength formalin–triethanolamine solution (7 ml formaldehyde + 2 ml triethanolamine + 71 ml distilled water), then mounted on glass microscope slides for taxonomic identification (Thorne & Allen, 1950; Seinhorst, 1959). Morphological observations and permanent slide preparations were performed using a Leica DM1000 compound microscope (4X-100X magnification). Identification of nematode specimens was carried out using polytomous keys (Loof & Luc, 1990).

Representative female specimens of *M. xenoplax*, after fixation, assumed a characteristic C-shaped body and were strongly annulated along their entire length. The lip region consisted of four distinct plates, with the first annule cup-shaped and slightly retracted. A chitinized valve was observed between the procorpus and metacorpus, and a well-developed median bulb and short isthmus surrounded by the nerve ring were noted. The excretory pore was located anteriorly, and the stylet was well developed with rounded basal knobs. The vulva was positioned near the posterior end, and the species was monodelphic with a single ovary extending toward the midbody. The anus was located just below the vulva and was often indistinct, while the tail terminus appeared rounded. Identification was based on morphological characters described by De Grisse (1969).

In contrast, female specimens of *X. pachtaicum* had a cylindrical body that tapered slightly toward the posterior end and typically appeared spiral or C-shaped after fixation. The lip region was rounded and clearly set off from the body by a shallow constriction. The cuticle was finely striated. The odontostyle was well developed, terminating with two distinct basal flanges, and the odontophore was slender and continuous with the guiding ring. The esophagus was cylindrical, with a basal bulb occupying approximately one-third of the body length, while the median bulb was absent. The vulva appeared slit-like and was located near the mid-body, with the vagina extending approximately one-third of the body width. The gonads were paired and opposed, and the prerectum was indistinct. The spermatheca was not observed, and a Z-organ was absent. The tail was short and conical, ending in a smooth, rounded terminus. Identification was performed according to Loof and Luc (1990) and Lamberti *et al*. (2000). Molecular confirmation was not included in this study, as the primary objective was to evaluate the accuracy of genus-level detection. Morphological identification was employed as the taxonomic reference standard to validate diagnostic accuracy before image annotation.

### Dataset Preparation

Microscopic images were manually annotated using MakeSense. AI, yielding 1,034 bounding box annotations for two nematode classes. *X. pachtaicum* (556 annotation) and *M. xenoplax* (478 annotation) were labeled as XIPHME and CRINXP following EPPO (2025) codes. In the dataset, 76 images contained non-target nematode taxa and served as background or negative samples. A total of 485 images included *X. pachtaicum*, either alone or co-occurring with other nematode species, while 400 images contained *M. xenoplax* individually or in association with additional taxa. In addition to the two target plant-parasitic nematodes, several non-target taxa listed in Table 1, representing bacterial-feeding, fungal-feeding, omnivorous, and predatory groups, were included to enhance generalization and species discrimination. Each image displays morphological identification features, including the lip region, vulva, tail, and entire body, to enable the models to learn and accurately distinguish nematode species from microscopic images. All images were standardized to 640 × 640 pixels within Roboflow before training. No data augmentation was applied to avoid altering key morphological features such as tail shape and stylet length. The datas*et al*ready included sufficient natural variation from multiple specimens, ensuring model generalization without artificial transformations. The dataset was divided into training (70 %), validation (20 %), and test (10 %) subsets. Model training and evaluation were conducted using Roboflow 3.0 (YOLOv8-compatible) with the YOLOv11 and YOLO-NAS Small architectures. The YOLO-based models were trained with standardized hyperparameters optimized for object detection. All models employed the Stochastic Gradient Descent (SGD) optimizer with a learning rate of 0.001, momentum of 0.937, and weight decay of 0.0005 to ensure stable convergence. A warm-up phase of three epochs was used to gradually ramp up the learning rate, followed by a total of 150 epochs for YOLO-NAS, 250 epochs for Roboflow 3.0 (YOLOv8 architecture), and 250 epochs for YOLOv11. The batch size was set to 8 – 16, and all input images were resized to 640 × 640 pixels. YOLOv11 and Roboflow 3.0 were implemented on the PyTorch framework, whereas YOLO-NAS utilized the AutoNAC optimization module for adaptive neural architecture configuration. Model training was performed entirely within the Roboflow platform using cloud-based computational resources powered by a PyTorch backend. Each model was trained multiple times independently to ensure optimal performance.

**Table 1.** Nematode species included in the training dataset for YOLO analysis.

*Xiphinema pachtaicum*	*Discolaimus*	*Paratylenchus nainianus*
*Acrobeloides nanus*	*Ditylenchus dipsaci*	*Pratylenchus thornei*
*Acrobeles cilliatus*	*Dorylaimus* sp.	*Psilenchus hilarulus*
*Alaimus primitivus*	*Filenchus thornei*	*Prodorylaimus* sp.
*Aphelenchus avenae*	*Geocenamus brevidens*	*Rhabditis* sp.
*Aphelenchoides sacchari*	*Mesocriconema xenoplax*	*Rotylenchus cypriensis*
*Boleodorus thylactus*	*Helicotylenchus multicinctus*	*Rotylenchulus macrosoma*
*Cephalobus persegnis*	*Helicotylenchus digonicus*	*Tylenchorhynchus cylindricus*
*Cervidellus* sp.	*Longidorus elongatus*	*Hoplolaimus galeatus*
*Clarkus papillatus*	*Mesodorylaimus* sp.	*Ditylenchus myceliophagus*

### Model Evaluation and Metrics

The performance of YOLO-based deep learning architectures in detecting *X. pachtaicum* and *M. xenoplax* was rigorously assessed through a combination of quantitative metrics and qualitative visual analyses. All models (YOLOv11, YOLO-NAS, and Roboflow 3.0 [YOLOv8 compatible]) utilized a total loss function (*L_total_*) consisting of localization, objectness, and classification components, which is characteristic of the YOLO architecture:


$$L_{\text{total }}=\lambda_{\text{box }}L_{\text{box }}+\lambda_{obj}L_{obj}+\lambda_{cls}L_{cls}$$

Here, *L_box_* denotes the localization loss, *L_obj_* represents the objectness confidence loss, and *L_cls_* refers to the classification loss. The weighting factors (*λ_box_, λ_obj_, λ_cls_*) were empirically adjusted to balance the contribution of each component to the total loss. In the YOLOv11 and Roboflow 3.0 models, the *L_box_* loss was calculated using CIoU (Complete Intersection over Union) or GIoU (Generalized Intersection over Union). At the same time, the *L*_obj_ and *Lcs* were computed using Binary Cross-Entropy (BCE) and Cross-Entropy, respectively. In contrast, YOLO-NAS employed a hybrid loss function that combined IoU (for localization), Focal Loss, and Cross-Entropy.

Model performances were evaluated using Precision, Recall, F1-score, and mean Average Precision (mAP) at IoU thresholds of 0.5 (mAP@50) and averaged from 0.5 to 0.95 (mAP@50-95). Confusion matrices were constructed to visualize inter-class misclassification rates and assess species-specific recognition accuracy. Prediction outcomes were classified into four categories based on comparison with ground truth annotations:

True Positives (TP): Correct detections of *X. pachtaicum* or *M. xenoplax*.

False Positives (FP): Incorrect detections where the model falsely labeled background or other objects as one of the target species.

False Negatives (FN): Missed detections where the model failed to identify a present target.

True Negatives (TN): Non-target regions accurately identified as negative.

From these outcomes, the following performance metrics were derived:

Accuracy (ACC) = (TP + TN) /(TP + TN + FP + FN)

→ Measures the overall proportion of correct predictions.


Precision (P) = TP / (TP + FP)

→Indicates how many of the predicted positives are actually correct.


Recall (R)= TP / (TP + FN)

→Indicates how many of the actual positives are correctly detected.


F1−Score= 2 × (P × R) / (P + R)

→Provides a balance between precision and recall. Mean Average Precision (mAP):


$$P=\frac{1}{N}\sum_{i}^{N}AP_{i}$$

→The sum of precision at each threshold multiplied by the change in recall between thresholds.

**Fig. 1.**
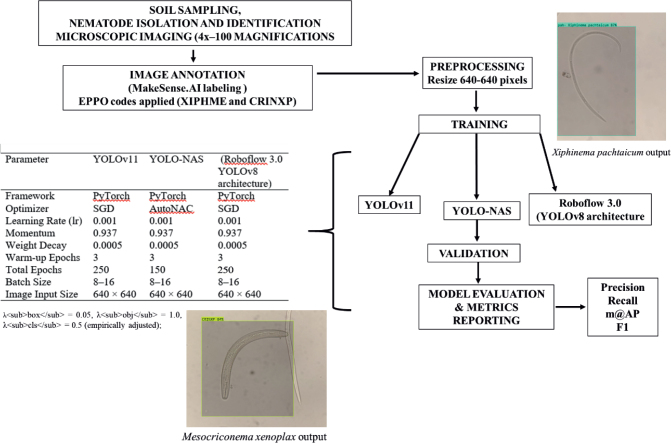
The Roboflow 3.0 (YOLOv8 architecture), YOLO-NAS, and YOLOv11 training workflow.

The Roboflow 3.0 (YOLOv8 architecture), YOLO-NAS, and YOLOv11 model workflow was represented in Figure 1.

## Results

**Fig. 2.**
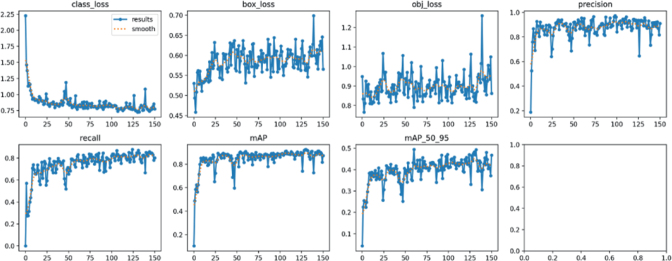
Training Loss Curves and Performance Metrics of YOLO-NAS.

The YOLO-NAS-based object detection model, trained on 961 labeled microscopic images, achieved high performance in identifying two nematode species: *Xiphinema pachtaicum* (XIPHME) and *Mesocriconema xenoplax* (CRINXP). The model reached a mean Average Precision at an IoU threshold of 0.5 (mAP@50) of 92.7 %. These metrics indicate a strong capacity to detect nematode samples, with minimal false positives and missed detections. Throughout approximately 150 training epochs, the model exhibited stable and effective learning behavior. Bounding box regression loss consistently decreased and stabilized between 0.45 and 0.70, reflecting improved localization accuracy of nematode specimens. Classification loss declined sharply in the early epochs and plateaued at around 0.6, while objectness loss fluctuated between 0.7 and 1.2, suggesting robust detection confidence (Fig. 2). Precision and recall curves converged early and remained consistent across epochs, further supporting the model’s reliability. The mAP@50 metric rose rapidly during the early training phase and plateaued at around 0.92, indicating strong overall detection performance. In contrast, the more stringent mAP@50-95 stabilized around 0.50, demonstrating moderate localization precision under tighter IoU thresholds.

**Fig. 3.**
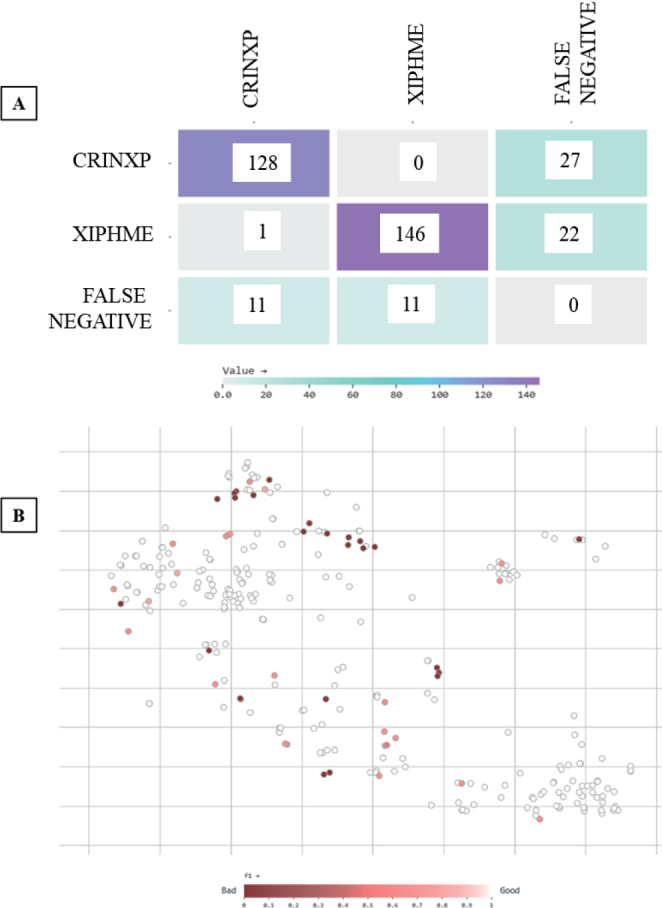
Evaluation outputs of YOLO-NAS (A): Confusion matrix of class predictions. (B) F1 score distribution across test images.

The confusion matrix (Fig. 3a) shows that the model made mostly correct predictions, correctly identifying 128 CRINXP and 146 XIPHME nematode images. However, it missed 27 CRINXP and 22 XIPHME cases, indicating that some nematode samples were not detected, possibly due to unclear morphology or overlapping features. Only one image was mislabeled as the other class, indicating that the model rarely confuses these two nematode types. There were also 11 false detections in background images for each class, indicating that the model generally performs well at separating nematodes from the background. To better understand how the model performed on each image, a vector-based analysis (Fig. 3b) was done. Each image was shown as a point in a two-dimensional space, with color indicating how well the model per-formed (based on the F1 score). Most images had high F1 scores (above 0.7), indicating the model performed well on them. A few clusters had low F1 scores (below 0.4) and were often grouped together in the plot. These were likely difficult samples, perhaps with low contrast, overlapping nematodes, or unclear boundaries.

**Fig. 4.**
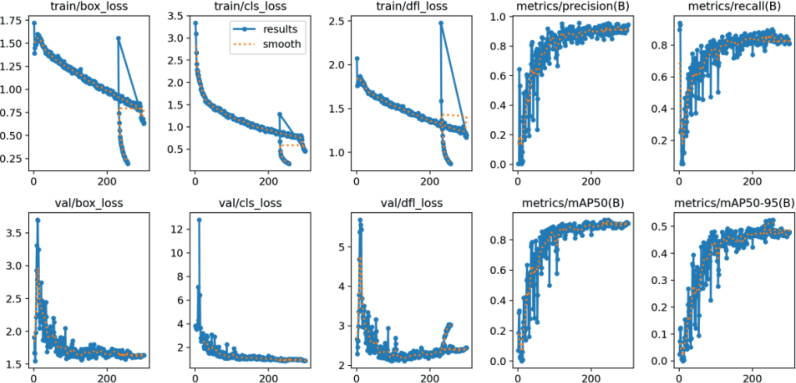
Training loss curves and performance metrics of YOLOv11.

The YOLOv11 object detection model, trained on a dataset of 961 annotated microscopic images, demonstrated strong performance in identifying nematode species. The model achieved a mean Average Precision at IoU 0.5 (mAP@50) of 93.2 %, indicating robust detection accuracy with minimal false positives. The training process spanned approximately 200 epochs, during which model learning dynamics were monitored via primary loss functions. The bounding box regression loss (box loss) steadily decreased and stabilized around 1.5, indicating enhanced spatial localization performance. The classification loss decreased sharply from initial values above 12 to below 2.0, reflecting the model’s increasing confidence in taxonomic assignments. Meanwhile, the objectness loss gradually decreased and plateaued at around 2.5, indicating improved object presence estimation across image regions. In terms of mean Average Precision over a range of IoU thresholds (mAP@50:95), the model showed continuous improvement throughout training and achieved a satisfactory final value, underscoring its generalization performance beyond fixed thresholds. The precision-recall curves demonstrated early convergence and consistency with final evaluation metrics, validating the model’s robustness across diverse image conditions (Fig. 4).

**Fig. 5.**
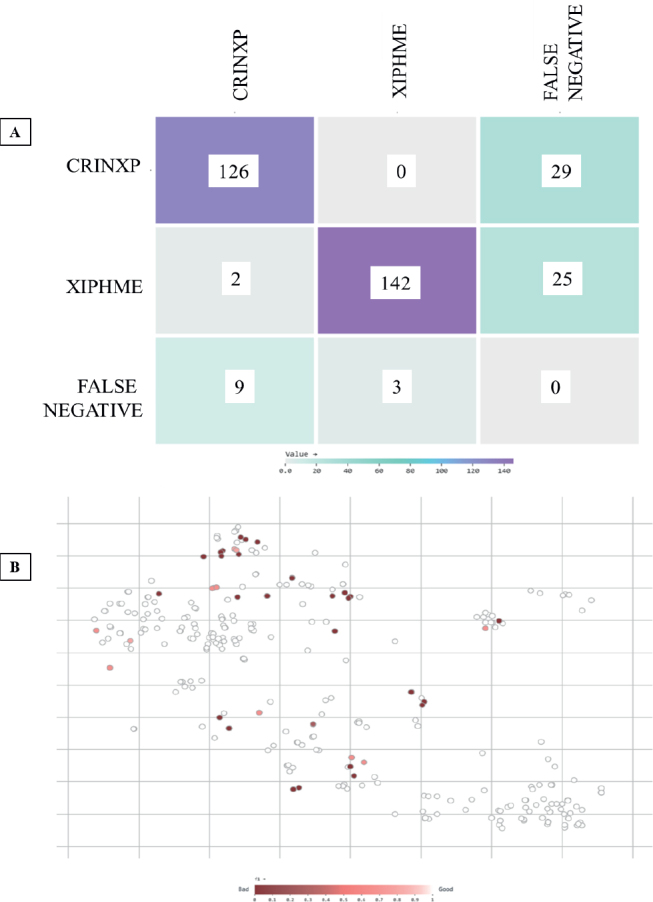
Evaluation outputs of YOLOv11 (A): Confusion matrix of class predictions. (B) F1 score distribution across test images.

Analysis of the confusion matrix (Fig. 5a) revealed that out of the test instances, the model correctly classified 126 images as CRINXP and 142 as XIPHME. Misclassifications were minimal, with only two instances of XIPHME erroneously identified as CRINXP, and no confusion in the opposite direction. False negatives accounted for 29 in CRINXP and 25 in XIPHME, while false positives were limited to 9 and 3 cases, respectively, further affirming the model’s specificity. The vector analysis map (Fig. 5b) revealed that the F1 scores of individual image embeddings were densely clustered within the F1 > 0.8 range, indicating consistent and high detection performance across the dataset.

**Fig. 6.**
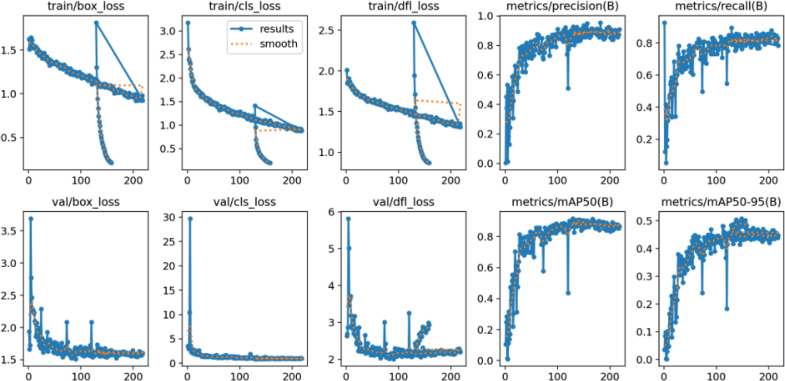
Training loss curves and performance metrics of Roboflow 3.0.

The Roboflow 3.0 (YOLOv8 compatible) model trained for nematode identification achieved a mean Average Precision at 0.5 IoU (mAP@50) of 89.4 %, indicating a high level of spatial agreement between predicted and ground-truth bounding boxes. The model also achieved a precision of 86.1 %, indicating a relatively low false-positive rate, and a recall of 82.7 %, suggesting a moderately strong ability to capture true instances in the annotated dataset. Training and validation curves confirmed effective learning dynamics over ~200 epochs: box loss steadily declined and stabilized around 1.5, class loss dropped below 1.0, and objectness loss converged between 0.8 and 1.0, all indicating improved localization, classification confidence, and object detection across the training process (Fig. 6).

**Fig. 7.**
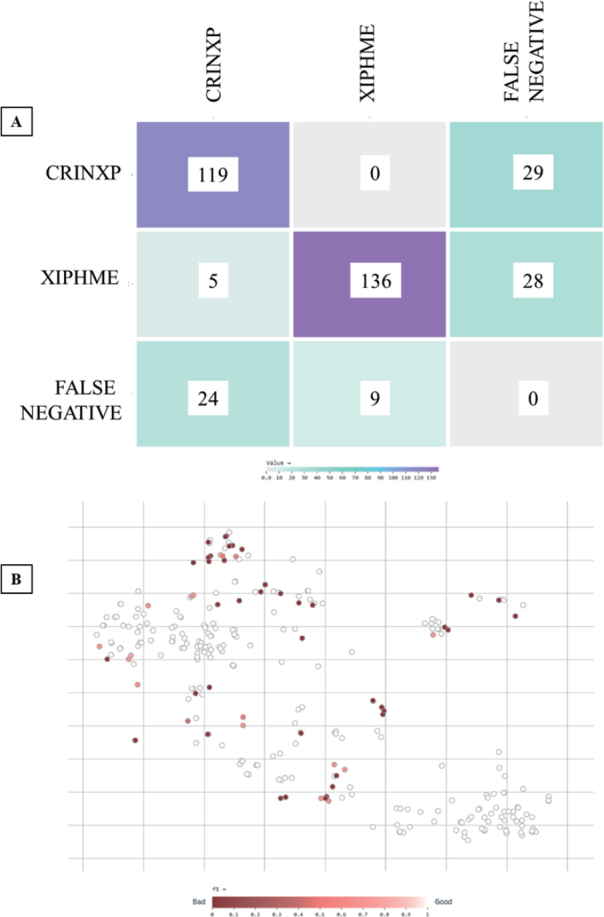
Evaluation outputs of Roboflow 3.0 (A): Confusion matrix of class predictions. (B) F1 score distribution across test images.

The confusion matrix (Fig. 7a) showed that the model correctly classified 119 images as CRINXP and 136 as XIPHME, with misclassification remaining relatively low: only 5 XIPHME cases were predicted as CRINXP and 28 XIPHME detections were missed. Background false positives were modest, totaling 24 for CRINXP and 9 for XIPHME, suggesting reasonable model specificity. The vector analysis map (Fig. 7b) further supported the model’s consistency: most image embeddings clustered in the F1-score range above 0.8, indicating stable detection confidence across test cases. Together, these results confirm that the Roboflow 3.0 model demonstrates robust classification accuracy and moderate localization precision, making it a reliable tool for object detection in nematode microscopy workflows, albeit with slightly lower detection strength compared to the YOLOv11.

## Discussion

In the present study, deep learning–based object detection models reliably detected *X. pachtaicum* and *M. xenoplax* at the genus level from high-resolution microscopic images. Classical nematode taxonomy relies on detailed morphometric measurements and expert-based morphological identification. Under routine laboratory conditions, large numbers of specimens may need to be examined within a limited time frame, and diagnostic accuracy can be influenced by workload, operator experience, and the quality of specimen preparation (Kumari & Lišková, 2009; Kumari *et al*., 2010). The high detection performance observed in this study indicates that YOLO-based architectures can consistently learn fine morphological traits, such as body curvature, annulation density, lip region configuration, and anterior morphology. These findings are consistent with previous studies reporting that deep neural networks can accurately capture subtle structural variations in microscopic biological specimens (Zaidi *et al*., 2022; Kaur & Singh, 2023). An Al-assisted system can help reduce dependence on individual expertise, support consistent decision-making, and provide rapid preliminary screening of samples before detailed taxonomic examination.

Among the evaluated models, YOLOv11 achieved the highest detection performance (mAP@50 = 93.2 %; precision = 95.7 %). It outperformed YOLO-NAS (mAP@50 = 92.7 %) and Roboflow 3.0 (mAP@50 = 89.4 %) in both localization accuracy and classification confidence. Performance improvements reported in recent YOLO models have been largely attributed to enhanced multi-scale feature fusion and improved sensitivity to small objects (Ahmad *et al*., 2022; Dai *et al*,. 2023; Gao *et al*,. 2025), which likely contributed to the results observed in this study.

The precision values for all trained models ranged from 86.1 % to 95.7 %. In microscopic preparations, specimens sometimes overlap, nematodes appear in different orientations during slide preparation, or show only partially visible, identifiable morphological structures. Variations in illumination and slide quality can further affect image clarity. In addition, the presence of morphologically similar species in the image dataset, such as *L. elongatus*, which shares certain structural features with *X. pachtaicum*, may increase classification difficulty. Although these morphological differences are readily distinguishable by an experienced taxonomist, such factors can slightly influence model confidence. Therefore, the observed precision of 95.7 % should be considered a strong and reliable performance under variable imaging conditions.

The inclusion of only two target genera does not imply a simplified dataset structure. On the contrary, the selected genera were derived from a nematode image dataset comprising 28 genera in total. This indicates that the model was trained within a morphologically diverse ecological context rather than an artificially reduced two-class scenario. The high accuracy values obtained under these conditions suggest that the model successfully learned genus-specific discriminative morphological characteristics.

YOLO-based approaches are increasingly being applied in nematology. Previous studies have demonstrated the successful use of YOLO architectures for detecting and quantifying root-knot nematodes (*Meloidogyne* spp.) (Pun *et al*., 2022; Wang *et al*., 2022). In particular, the YOLOv5-based NemDST system developed by Pun *et al*. (2023) achieved 0.99 precision and 0.97 mAP in the automatic detection of root-knot nematode eggs. However, these studies primarily focused on egg detection or population estimation. The present study extends the application of YOLO architectures to genus-level taxonomic discrimination between two morphologically similar plant-parasitic nematodes. YOLOv11’s ability to distinguish subtle inter-genus morphological differences further demonstrates the increasing taxonomic resolution capacity of newer-generation detection models.

Given the small size and high morphological similarity of plant-parasitic nematodes, architectural design plays a critical role in detection performance. In YOLOv11, C3k2 blocks improve computational efficiency, while the C2PSA spatial attention module enables the model to focus on diagnostically relevant regions, such as the anterior morphology, body curvature, and annulation structures. This attention-driven feature extraction may enhance discrimination in microscopic images characterized by low contrast, partial occlusion, or background debris. Achieving high mAP values with a relatively compact parameter structure also suggests an efficient internal feature representation (Khanam & Hussain, 2024; Sapkota *et al*,. 2025). In contrast, YOLO-NAS achieves performance gains primarily through an automated architecture search strategy. Its Neural Architecture Search (NAS)–based optimization framework systematically balances detection accuracy and inference speed. The integration of quantization-aware design and INT8 compatibility provides practical advantages for portable microscopy systems and resource-constrained field devices, thereby increasing its suitability for real-time applications (Gupta *et al*., 2025).

Furthermore, comparative studies on high-resolution industrial inspection tasks show that single-stage CNN-based detectors often perform more stably than transformer-based architectures when detecting small objects under complex visual conditions. In particular, CNN models trained on large-scale, augmented datasets have been reported to achieve high mAP and recall while maintaining low error rates. These observations are relevant to nematode detection, where fine structural details at the microscopic scale are decisive. The results obtained in the present study therefore support the suitability of convolutional feature hierarchies for fine-grained morphological recognition, particularly in scenarios with limited object size and high background variability (Aleman & Duke, 2025).

In conclusion, YOLOv11 enhances morphological discrimination through attention-based architectural refinements, whereas YO-LO-NAS provides advantages in hardware efficiency and inference optimization. In applications such as nematode detection, which require both high diagnostic precision and practical deployability, model selection should be guided by a balanced consideration of accuracy and computational efficiency.

## Conclusions

The comparative analysis of YOLO-NAS, YOLOv11, and Roboflow 3.0 confirms the potential of deep learning for *X. pachtaicum* and *M. xenoplax* nematode identification. YOLOv11 stood out with the highest precision and accuracy, ideal for precise diagnostics. YO-LO-NAS offered strong recall and adaptability, making it well suited to variable imaging conditions and real-time use. Roboflow 3.0, while slightly less accurate, remains practical for fast deployment and integration. These results highlight the value of YOLO-based models in enhancing nematode detection workflows.
